# Apolipoprotein E Gene Polymorphisms Are Associated with Primary Hyperuricemia in a Chinese Population

**DOI:** 10.1371/journal.pone.0110864

**Published:** 2014-10-30

**Authors:** Jie Wu, Ling Qiu, Xiu-zhi Guo, Tao Xu, Xin-qi Cheng, Lin Zhang, Peng-chang Li, Qian Di, Qing Wang, Lan Ni, Guang-jin Zhu

**Affiliations:** 1 Department of Clinical Laboratory, Peking Union Medical College Hospital, Peking Union Medical College & Chinese Academy of Medical Science, Beijing, China; 2 Department of Epidemiology and Statistics, Institute of Basic Medical Sciences, Chinese Academy of Medical Sciences & Peking Union Medical College, Beijing, China; 3 Medical Experimental Center, General Hospital of Ningxia Medical University, Yinchuan, China; 4 Department of Pathophysiology, Institute of Basic Medical Sciences, Chinese Academy of Medical Sciences & Peking Union Medical College, Beijing, China; National Cancer Center, Japan

## Abstract

**Objective:**

Primary hyperuricemia, an excess of uric acid in the blood, is a major public health problem. In addition to the morbidity that is attributable to gout, hyperuricemia is also associated with metabolic syndrome, hypertension, and cardiovascular disease. This study aims to assess the genetic associations between Apolipoprotein E (APOE) polymorphisms and hyperuricemia in a Chinese population.

**Methods:**

A total of 770 subjects (356 hyperuricemic cases and 414 normouricemic controls) were recruited from the Ningxia Hui Autonomous Region, China. A physical examination was performed and fasting blood was collected for biochemical tests, including determination of the levels of serum lipid, creatinine, and uric acid. Multi-ARMS PCR was applied to determine the APOE genotypes, followed by an investigation of the distribution of APOE genotypes and alleles frequencies in the controls and cases.

**Results:**

The frequencies of the APOE-ε2ε3 genotype (17.70% vs. 10.39%, *P* = 0.003) and the APOE-ε2 allele (10.53% vs. 5.80%, *P* = 0.001) were significantly higher in the hyperuricemic group than in the normouricemic group. Furthermore, male cases were more likely to have the APOE-ε2ε3 genotype and APOE-ε2 allele, compared with male controls. In both Han and Hui subjects, cases were more likely to have the APOE-ε2ε3 genotype and the APOE-ε2 allele compared with controls. Furthermore, multivariate logistic regression showed that carriers of the APOE-ε2ε3 genotype (*P* = 0.001, OR = 2.194) and the ε2 allele (*P* = 0.001, OR = 2.099) were significantly more likely to experience hyperuricemia than carriers of the ε3/ε3 genotype and the ε3 allele after adjustment for sex, body mass index (BMI), diastolic blood pressure (DBP), triglyceride (TG), low density lipoprotein cholesterol (LDL-C), creatinine (Cr) and fasting blood glucose(FBG).

**Conclusions:**

The APOE-ε2ε3 genotype and the APOE-ε2 allele are associated with serum uric acid levels in Chinese subjects, indicating that individuals carrying the APOE-ε2 allele have a higher risk of hyperuricemia than non-carriers.

## Introduction

Serum uric acid (SUA) is the final product of purine metabolism in humans. Hyperuricemia(HUA), which is caused by the overproduction of urate or, more commonly, by the reduced renal excretion of urate, contributes to the development of gout [1]. It was reported that 18.8% of patients with HUA developed gout in a 5-year follow-up [2]. Elevation of the SUA level has been identified as a potential risk factor for developing hypertension, insulin resistance, dyslipidemia and obesity, which are hallmarks of metabolic syndrome [3]–[6]. In addition, multiple epidemiologic studies have confirmed an association between hyperuricemia and cardiovascular disease (CVD) [7]–[9]. The increasing occurrence of hyperuricemia was observed not only in western populations but also in Asia, including China. Our previous study has shown that the prevalence of HUA is 13.7% in northern China [10]. Recently a meta-analysis demonstrated that in China, the prevalence of hyperuricemia was 21.6% in males and 8.6% in females [11].

The apolipoprotein E (APOE) gene, located in the 19q13 chromosomal region, encodes the ApoE protein, which plays an important role in transporting cholesterol and other lipoproteins. The APOE gene has three common alleles, designated as ε2, ε3, ε4, and six different genotypes, ε2/2, ε2/3, ε2/4, ε3/3, ε3/4, ε4/4. These three common alleles result from two single-base polymorphisms within exon 4, at codons 112 and 158[12]. APOE-ε3 has a cysteine at residue 112 and an arginine at 158, which are required for normal ApoE functioning. APOE-ε2 (Arg158→Cys) has been shown to be associated with decreased levels of total and low density lipoprotein (LDL) cholesterol, whereas APOE-ε4 (Cys112→Arg) is associated with increased cholesterol levels [13], [14].

In addition to the impact on the serum lipid profile, blood pressure levels [15], the occurrence and development of type 2 diabetes mellitus [16] and neurodegenerative disorders [17], polymorphisms of the APOE gene are also associated with the risk of cardiovascular diseases [18]. An association between APOE polymorphisms and SUA was also demonstrated in several studies. However, other studies have reported inconsistent results. Some studies suggest that the ε2 allele is independently associated with increased SUA levels [19], [20] and that ε4 is associated with decreased SUA levels [21], [22], but others show that the ε4 allele is associated with a higher risk of hyperuricemia [23]. Possible causes include racial and ethnic differences. At present, the correlation between APOE polymorphisms and hyperuricemia remains elusive in China, especially among individuals in an area inhabited by ethnic minorities.

Therefore, the objective of this study was to assess the genetic associations of APOE polymorphisms and hyperuricemia in a Chinese population.

## Methods

### Subjects

The subjects were selected from a population-based cross-sectional survey, the Chinese Physiological Constant and Health Condition (CPCHC), in the Ningxia Hui Autonomous Region (2011). Briefly, this survey included 6056 participants, aged 10 to 80 years, who underwent physical examination and assays of serum biochemical indicators, including the serum urate levels. Of these 6056 subjects, 356 participants were initially screened as hyperuricemic cases in the present study. One or two region-, gender- and age-matched (the age difference did not exceed 2 years) normouricemic controls for each case were randomly selected at the time of the case diagnosis. Hyperuricemia was defined as SUA≥7 mg/dl (416 µmol/L, male) or SUA≥6 mg/dl (357 µmol/L, female), which is a widely accepted diagnostic criteria [24]–[26]. Finally, a total of 356 hyperuricemic subjects (243 male and 113 female) and 414 normouricemic individuals (218 male and 196 female) were recruited for genetic analysis. Participants with known systemic diseases, including diabetes mellitus, hypertension, cardiovascular disease, renal or liver disease, gastrointestinal disease, pulmonary disease, or cancer were excluded. Moreover, participants taking any medication known to affect carbohydrate and lipid metabolism were also excluded. The study was conducted in accordance with the Helsinki Declaration and was approved by the Ethics Committee of the Institute of Basic Medical Sciences at the Chinese Academy of Medical Sciences. Written informed consent was obtained from each adult participant before data collection. For minors/children, we sent the parents/guardians an information sheet about the study and obtained their written informed consent on behalf of the minors/children enrolled in our study.

### Clinical Laboratory Tests

All procedures were performed following a 9–12 hour overnight fast and all subjects were told to consume a bland diet before blood testing. Blood pressure (BP) was measured using an OMRON HEM-7000 electronic sphygmomanometer (OMRON HealthCare, Kyoto, Japan) after the participant had rested for ≥10 min. Blood was drawn from the antecubital vein of the arm. Total cholesterol (TC), triglycerides (TG), high-density lipoprotein cholesterol (HDL-C) and low-density lipoprotein cholesterol (LDL-C) were measured with a Beckman AU Series Automatic Biochemical Analyzer (Japan), using Sekisui Medical (Japan) reagents. Fasting blood glucose (FBG), uric acid (UA), creatine (Cr), and blood urea nitrogen (BUN) were measured with the same instrument, using Beckman AU reagents. HbA1c was examined by high-performance liquid chromatography (HPLC) on a Bio-Rad Diamat automated glycosylated hemoglobin analyzer (USA).

### APOE Genotyping

Genomic DNA for APOE genotyping was extracted from peripheral blood mononuclear cells with the Genomic DNA Purification System (Promega, Madison, WI, USA). The DNA samples were then analyzed using the amplification refractory mutation system (ARMS)-polymerase chain reaction (PCR) assay [27]. Briefly, the PCR was conducted with the following primers: Cys158/Arg158 (5′-ATGCCGATGACCTGCAGAATT-3′)/(5′ATGCCGATGACCTGCAGAATC-3′), Cys112/Arg112(5′-CGCGGACATGGAGGACGTTT-3′)/(5′-CGCGGACATGGAGGACGTTC-3′), and a common reverse primer (5′-GTTCAGTGATTGTCGCTGGGCA-3′). The common primer was paired with Cys158/Arg158 or Cys112/Arg112 and produced an amplicon of 451 and 588 base pairs (bp), respectively. A 218-bp fragment of the LDLR gene was co-amplified to function as an internal positive control, and the PCR primer sequences were 5′- GTGGCACCGAGACCAAACTC-3′ and 5′- AGTGCAAGGAGACCACGGGA-3′. The PCR conditions were denaturation at 95°C for 5 min, followed by 35 cycles of 95°C for 30 sec, 63°C for 30 sec and 72°C for 30 sec, and a final extension at 72°C for 5 min. At the end of the PCR cycles, the products were resolved by electrophoresis on 2% agarose gels to validate the amplification of the specific PCR product expected.

### Statistical Analysis

Continuous variables with a normal distribution are given as the mean ± standard deviation (SD) and were analyzed by t-test. Variables with a non-normal distribution are given as the median (interquartile range) and were compared by the Wilcoxon rank sum test. Categorical variables (such as ethnicity, genotype distribution and allele frequency) were analyzed as percentages using the Chi-squared test. The extremely rare genotype groups (ε2/ε2, ε4/ε4 and ε2/ε4) were excluded from the genotype analyses, but they were included in the allele analyses. ANOVA or the Wilcoxon rank sum test were used to compare the data related to the clinical parameters among the different APOE genotypes. Genotype and alleles frequencies for APOE were calculated and compared between the hyperuricemia cases and the control subjects using the Chi-squared and Fisher’s exact test. The associations between the ApoE genotypes and alleles and hyperuricemia were measured by calculating odd ratios (ORs) and 95% confidence intervals (CIs) using logistic regression. Univariate logistic analysis was applied to examine association between each potential risk factor and HUA. In multivariate analyses, all factors that were statistically significant were adjusted as potential confounding variables and the adjusted ORs and 95% CIs were calculated. Hardy-Weinberg equilibrium was assessed using the chi-squared test. In each model, the homozygous ε3/ε3 genotypes formed the reference group. Statistical significance was assumed at *P*<0.05, and all statistical analyses were conducted using SPSS.

## Results

### Clinical characteristics of the normouricemic and hyperuricemic groups stratified by gender

As shown in Table 1, there was no significant difference in mean age between the normouricemic group and the hyperuricemic group for either men or women. Regardless of gender, the hyperuricemic group had significantly higher values for body mass index (BMI), waist circumference (WC), systolic blood pressure (SBP), total cholesterol (TG), fasting blood glucose FBG, Glycated hemoglobin (HbA_1C_), uric acid (UA), creatine (Cr) and blood urea nitrogen (BUN), but they had lower high-density lipoprotein cholesterol (HDL-C) levels compared with the normouricemic group (*P*<0.05). The levels of Total cholesterol (TC) and low-density lipoprotein cholesterol (LDL-C) were significantly higher among women in the hyperuricemic group than in the normouricemic group, while the diastolic blood pressure (DBP) was significantly higher for men in the hyperuricemic group than in the normouricemic group.

**Table 1:** Demographic information and blood chemistry parameters of study subjects.

	Male(n = 461)		Female(n = 309)	
	Normouricemic group	Hyperuricemic group	Normouricemic group	Hyperuricemic group
N(%)	218(47.3%)	243(52.7%)	196(63.4%)	113(36.6%)
Age (years)	24.3(13.9–48.6)	24.0(15.8–45.5)	17.3(15.2–39.1)	18.0(13.2–57.2)
Ethnicity (%)				
Han	103(47.2%)	143(58.8%)	108(55.1%)	63(55.8%)
Hui	112(51.4%)	97(39.9)	88(44.9%)	48(42.5%)
BMI (kg/m^2^)	23.0±4.8	24.6±4.5*	21.6±3.5	23.5±5.1*
WC (cm)	76.8±14.2	80.1±12.5*	71.3±10.1	74.3±12.8*
SBP (mmHg)	126.7±21.0	133.3±18.8*	121.3±17.8	127.6±24.2*
DBP (mmHg)	77.4±13.9	82.5±14.7*	76.2±11.6	79.3±14.3
TC (mmol/L)	3.95±0.86	4.11±0.99	3.95±0.84	4.35±1.17*
TG (mmol/L)	1.08(0.65–1.72)	1.33(0.76–2.39)*	0.94(0.58–1.30)	1.20(0.81–1.96)*
HDL-C (mmol/L)	1.13±0.25	1.08±0.28*	1.27±0.27	1.20±0.27*
LDL-C (mmol/L)	2.20(1.76–2.69)	2.34(1.84–2.92)	2.18(1.73–2.61)	2.51(2.03–3.06)*
FBG (mmol/L)	4.96(4.68–5.33)	5.13(4.90–5.51)*	4.87(4.61–5.18)	5.09(4.87–5.52)*
HbA_1C_ (%)	5.10(4.90–5.40)	5.20(5.00–5.50)*	5.10(4.90–5.30)	5.20(5.00–5.60)*
UA (µmmol/L)	297.6±63.0	460.4±45.4*	239.3±55.6	398.4±40.0*
Cr (mmol/L)	80.37±15.4	88.99±18.6*	66.93±8.8	73.03±13.4*
BUN (mmol/L)	4.57±1.29	4.92±1.47*	3.96±1.16	4.47±1.27*

Abbreviations: BMI, Body mass index; WC, Waist circumference; SBP, Systolic blood pressure; DBP, Diastolic blood pressure; TC, Total cholesterol; TG, Triglyceride; HDL-C, High density lipoprotein cholesterol; LDL-C, Low density lipoprotein cholesterol; FBG, Fasting blood glucose; HbA_1C_, Glycated hemoglobin; UA, Uric acid; Cr, Creatinine; BUN, Blood urea nitrogen.

Data are presented as n (%), mean ± standard deviation (SD); Age, TG, LDL-C, FBG and HbA_1C_ were reported as the medians (interquartile range).

*P<0.05 for Hyperuricemic group vs. Normouricemic group within the same sex.

### Distribution of APOE genotypes and alleles among the study subjects

A total of 356 hyperuricemic cases and 414 normouricemic subjects were evaluated, and the frequencies of the APOE genotypes and alleles are shown in Table 2. The allele frequencies were within Hardy-Weinberg equilibrium (*P*>0.05). The frequencies of the ε2ε3 genotype and the ε2 allele in the hyperuricemic group were significantly higher than those in the normouricemic group (*P* = 0.003 and *P* = 0.001, respectively). However, the distributions of the ε3ε3 genotype and the ε3 allele were significantly lower in the hyperuricemic individuals (*P* = 0.008 and *P* = 0.007, respectively).

**Table 2:** Genotype and allele frequencies of APOE polymorphisms among the study subjects.

	Normouricemic group (%)	Hyperuricemic group (%)	*P value*
	(n = 414)	(n = 356)	
Genotypes			
ε2ε2	1(0.24%)	3(0.84%)	
ε2ε3	43(10.39%)	63(17.70%)	0.003
ε2ε4	3(0.72%)	6(1.69%)	
ε3ε3	304(73.43%)	230(64.61%)	0.008
ε3ε4	60(14.49%)	52(14.61%)	0.522
ε4ε4	3(0.72%)	2(0.56%)	
Alleles			
ε2	48(5.80%)	75(10.53%)	0.001
ε3	711(85.87%)	575(80.76%)	0.007
ε4	69(8.33%)	62(8.71%)	0.431

The allelic and genotypic frequencies are indicated in absolute values and percentage. *P* value for the overall comparison between the hyperuricemic and normouricemic groups by the chi-square test.

The extremely rare genotypes-ε2ε2, ε2ε4 and ε4ε4 subjects were described but excluded from the statistical analysis.

### Distribution of APOE genotypes and alleles based on gender and ethnicity

Table 3 and 4 present the APOE genotype and allele frequencies distributed by gender and ethnic group, respectively. The ε2ε3 genotype frequency in the hyperuricemic group was significantly higher than that in the normouricemic group for males (*P* = 0.039). Furthermore, the APOE ε2 allele frequency was significantly higher in the hyperuricemic group compared with the normouricemic group both in males (*P* = 0.014) and females (*P* = 0.049). In addition, the frequencies of the APOE-ε3ε3 genotype and the ε3 allele in the hyperuricemic group were significantly lower than those in the normouricemic group in males but not in females. Meanwhile, we observed that the prevalence of the APOE ε2 allele in males was higher than that in females in both the hyperuricemic (11.3% vs. 8.8%) and the normouricemic (6.7% vs. 4.8%) groups, although the difference was not statistically significant. Similar results were obtained from different ethnic groups (Table 4). The frequencies of the ε2ε3 genotype and the ε2 allele were significantly higher, but those of the ε3ε3 genotype and the ε3 allele were significantly lower in the hyperuricemic group compared with the normouricemic group in the Han population. Moreover, in the Hui population, a significant difference in the distribution of the ε2 allele between the control and hyperuricemic groups was identified.

**Table 3:** The APOE genotype and allele frequencies among the study subjects stratified by gender.

	Male		Female		*P-value* a	*P-value* b
	Normouricemic group	Hyperuricemic group	Normouricemic group	Hyperuricemic group		
Genotypes						
ε2ε2	1(0.5%)	2(0.8%)	0(0%)	1(0.9%)		
ε2ε3	26(11.9%)	46 (18.9%)	17(8.7%)	17(15.0%)	0.039	0.064
ε2ε4	1(0.5%)	5(2.1%)	2(1.0%)	1(0.9%)		
ε3ε3	160(73.4%)	154(63.4%)	144(73.5%)	76(67.3%)	0.021	0.245
ε3ε4	28(12.8%)	34(14.0%)	32(16.3%)	18(15.9%)	0.718	0.927
ε4ε4	2(0.9%)	2(0.8%)	1(0.5%)	0(0%)		
Total	218	243	196	113		
Alleles						
ε2	29(6.7%)	55(11.3%)	19(4.8%)	20(8.8%)	0.014	0.049
ε3	374(85.8%)	388(79.8%)	337(86.0%)	187(82.7%)	0.017	0.282
ε4	33(7.6%)	43(8.8%)	36(9.2%)	19(8.4%)	0.481	0.744
Total	436	486	392	226		

Subjects with the extremely rare genotypes -ε2ε2, ε2ε4 and ε4ε4 were described but excluded from the statistical analysis.

a Hyperuricemic group *vs.* Normouricemic group in males.

b Hyperuricemic group *vs.* Normouricemic group in females.

**Table 4:** The APOE genotype and allele frequencies among the study subjects stratified by ethnic group.

	Han		Hui		*P-value* a	*P-value* b
	Normouricemic group	Hyperuricemic group	Normouricemic group	Hyperuricemic group		
Genotypes						
ε2ε2	1(0.5%)	1(0.5%)	0(0%)	2(1.4%)		
ε2ε3	20(9.5%)	36(17.5%)	23(11.5%)	27(18.6%)	0.017	0.064
ε2ε4	3(1.4%)	3(1.5%)	0(0%)	3(2.1%)		
ε3ε3	158(74.9%)	131(63.6%)	143(71.5%)	95(65.5%)	0.012	0.236
ε3ε4	28(13.3%)	34(16.5%)	32(16%)	17(11.7%)	0.353	0.261
ε4ε4	1(0.5%)	1(0.5%)	2(1%)	1(0.7%)		
Total	211	206	200	145		
Alleles						
ε2	25(5.9%)	41(10.0%)	23(5.8%)	34(11.7%)	0.021	0.005
ε3	364(86.3%)	332(80.6%)	341(85.3%)	234(80.7%)	0.027	0.113
ε4	33(7.8%)	39(9.5%)	36(9%)	22(7.6%)	0.397	0.509
Total	422	412	400	290		

Subjects with the extremely rare genotypes -ε2ε2, ε2ε4 and ε4ε4 were described but excluded from the statistical analysis.

a Hyperuricemic group *vs.* Normouricemic group in Han.

b Hyperuricemic group *vs.* Normouricemic group in Hui.

### Effect of APOE gene polymorphism on metabolic parameters and serum uric acid concentrations

We further analyzed the effects of the APOE genotypes on the clinical and metabolic parameters and serum uric acid levels in both the normouricemic group and hyperuricemic group. As shown in Table 5, the levels of TC, LCL-C and UA were significantly different among the APOE genotype groups in both the normouricemic group and hyperuricemic group. The ε2/3 group had significantly higher levels of serum uric acid compared with the ε3/3 group (P<0.05).

**Table 5:** The effects of APOE genotypes on clinical and metabolic parameters.

	Normouricemic group			Hyperuricemic group			*P-value* *	*P-value* #
	ε2/3(n = 43)	ε3/3(n = 304)	ε3/4(n = 60)	ε2/3(n = 63)	ε3/3(n = 230)	ε3/4(n = 52)		
Age (years)	22.6(15.0–44.2)	22.1(14.9–45.4)	19.7(13.0–43.7)	19.0(14.0–47.1)	21.0(15.7–51.4)	19.1(13.2–43.4)	0.916	0.225
BMI(kg/m^2^)	22.4±3.86	22.3±4.31	22.0±4.21	24.5±5.12	24.1±4.68	24.2±4.44	0.879	0.898
WC(cm)	74.6±11.3	74.2±12.7	73.1±13.6	79.1±13.1	77.8±12.9	78.7±11.3	0.808	0.749
SBP	122.9±20.1	124.3±19.3	124.3±22.0	133.8±21.3	132.2±21.3	135.9±17.4	0.920	0.801
DBP	75.1±12.9	76.9±12.9	77.3±12.6	83.2±15.4	80.9±14.9	81.8±13.3	0.666	0.547
TC(mmol/L)	3.68±0.75^ab^	3.98±0.87	4.01±0.80	3.81±1.09^ab^	4.27±1.04	4.35±1.00	0.049	0.005
TG(mmol/L)	0.84(0.53–1.28)	0.99(0.62–1.51)	1.05(0.62–1.52)	1.35(0.88–2.68)	1.21(0.73–2.13)	1.53(0.97–2.58)^C^	0.327	0.054
HDL-C(mmol/L)	1.27±0.28	1.20±0.27	1.19±0.27	1.11±0.27	1.13±0.29	1.05±0.20	0.219	0.105
LDL-C(mmol/L)	1.89(1.45–2.38)^ab^	2.21(1.76–2.71)	2.18(1.87–2.57)	1.83(1.53–2.35)^ab^	2.45(2.01–3.06)	2.68(2.22–3.16)	0.005	<0.001
FBG(mmol/L)	4.85(4.59–5.22)	4.93(4.65–5.25)	4.92(4.60–5.22)	5.14(4.97–5.58)	5.12(4.89–5.49)	5.13(4.67–5.59)	0.536	0.349
HbA_1C_ (%)	5.10(5.00–5.30)	5.10(4.90–5.30)	5.10(4.80–5.40)	5.20(5.10–5.50)	5.20(5.00–5.50)	5.20(5.00–5.60)	0.932	0.922
UA(µmol/L)	283.7±57.3^a^	264.9±57.4	273.2±52.8	448.4±54.4^a^	432.4±52.1	437.2±52.4	0.039	0.024
Cr (mmol/L)	75.1±13.6	74.2±14.9	72.2±12.4	83.2±17.6	84.6±18.9	81.0±18.5	0.540	0.430
BUN (mmol/L)	4.25±1.31	4.32±1.22	4.18±1.31	4.52±1.44	4.87±1.44	4.61±1.36	0.741	0.168

Abbreviations: BMI, Body mass index; WC, Waist circumference; SBP, Systolic blood pressure; DBP, Diastolic blood pressure; TC, Total cholesterol; TG, Triglyceride; HDL-C, High density lipoprotein cholesterol; LDL-C, Low density lipoprotein cholesterol; FBG, Fasting blood glucose; HbA_1C_, Glycated hemoglobin; UA, Uric acid; Cr, Creatinine; BUN, Blood urea nitrogen.

LSD and SNK test were applied to analyze the differences between each pair of groups.

a ε2/3 vs. ε3/3, p<0.05;

b ε2/3 vs. ε3/4, p<0.05;

C ε3/4 vs. ε3/3, p<0.05.

*Comparison of genotypes in Normouricemic group.

# Comparison of genotypes in Hyperuricemic group.

Table 6 presents the association between the presence of APOE gene polymorphism, clinical metabolic parameters and hyperuricemia. The results of univariate logistic regression revealed that carriers of the ε2ε3 genotype (*P* = 0.002, OR = 1.937, 95% CI 1.267–2.959) and ε2 allele (*P* = 0.001, OR = 1.932, 95% CI 1.323–2.821) were significantly more likely to present hyperuricemia when the ε3/ε3 genotypes was set as the reference group. Furthermore, sex, BMI, WC, SBP, DBP, TC, TG, HDL-C, LDL-C, BUN, Cr, FBG and HbA_1C_ were found significantly associated with hyperuricemia. Considering the high degree of correlation between some variables (for example, SBP and DBP), in the next multivariate logistic regression model, only key factors sex, BMI, DBP, TG, LDL-C, Cr and FBG were included and adjusted as potential confounding variables. As shown in Table 7, the ε2ε3 genotype (*P* = 0.001, OR = 2.194, 95% CI 1.362–3.537) and ε2 allele (*P* = 0.001, OR = 2.099, 95% CI 1.379–3.195) were also significantly associated with hyperuricemia after adjusting for sex, BMI, DBP, TG, LDL-C, Cr and FBG.

**Table 6:** Univariate analysis of the association between APOE polymorphism, metabolic parameters and hyperuricemia.

	*OR (95%CI)*	*P value*
Genotypes		
ε3ε3	1(Reference)	
ε2ε3	1.937(1.267–2.959)	0.002
ε3ε4	1.146(0.761–1.724)	0.515
Alleles		
ε3	1(Reference)	
ε2	1.932(1.323–2.821)	0.001
ε4	1.111(0.775–1.593)	0.567
Sex		
Female	1(Reference)	
Male	1.933(1.439–2.597)	<0.001
Age(years)	1.006(0.998–1.013)	0.129
BMI(kg/m^2^)	1.097(1.061–1.133)	<0.001
WC(cm)	1.025(1.014–1.037)	<0.001
SBP	1.018(1.010–1.025)	<0.001
DBP	1.025(1.014–1.036)	<0.001
TC(mmol/L)	1.299(1.116–1.512)	0.001
TG(mmol/L)	1.534(1.325–1.776)	<0.001
HDL-C(mmol/L)	0.342(0.200–0.585)	<0.001
LDL-C(mmol/L)	1.419(1.167–1.726)	<0.001
FBG(mmol/L)	1.648(1.303–2.084)	<0.001
HbA_1C_ (%)	2.052(1.447–2.910)	<0.001
Cr(mmol/L)	1.040(1.029–1.050)	<0.001
BUN(mmol/L)	1.316(1.179–1.469)	<0.001

Abbreviations: BMI, Body mass index; WC, Waist circumference; SBP, Systolic blood pressure; DBP, Diastolic blood pressure; TC, Total cholesterol; TG, Triglyceride; HDL-C, High density lipoprotein cholesterol; LDL-C, Low density lipoprotein cholesterol; FBG, Fasting blood glucose; HbA_1C_, Glycated hemoglobin; Cr, Creatinine; BUN, Blood urea nitrogen.

*P*-values and their ORs for the genotypes and clinical parameters were calculated by univariate logistic regression analysis.

**Table 7:** Multivariate analysis of the independent association between APOE polymorphism and hyperuricemia.

	*OR (95%CI)*	*P value*
Genotypes		
ε3ε3	1(Reference)	
ε2ε3	2.194(1.362–3.537)	0.001
ε3ε4	1.175(0.755–1.828)	0.475
Alleles		
ε3	1(Reference)	
ε2	2.099(1.379–3.195)	0.001
ε4	1.076(0.730–1.588)	0.710
Sex		
Female	1(Reference)	
Male	1.093(0.757–1.579)	0.635
BMI(kg/m^2^)	0.988(0.940–1.037)	0.621
DBP	1.000(0.986–1.014)	0.957
TG(mmol/L)	1.227(1.034–1.455)	0.019
LDL-C(mmol/L)	1.149(0.891–1.481)	0.285
FBG(mmol/L)	1.378(1.060–1.792)	0.017
Cr(mmol/L)	1.033(1.018–1.047)	<0.001

Abbreviations: BMI, Body mass index; DBP, Diastolic blood pressure; TG, Triglyceride; LDL-C, Low density lipoprotein cholesterol; FBG, Fasting blood glucose; Cr, Creatinine.

P -values and adjusted ORs for the multivariate logistic regression analyses were adjusted for sex, BMI, DBP, TG, LDL-C, FBG and Cr.

## Discussion

In the present study we observed that APOE gene polymorphism is associated with hyperuricemia in Chinese subjects. The frequencies of both the APOE-ε2ε3 genotype and ε2 allele were higher in the hyperuricemic group than in the normouricemic group (p<0.01), and individuals with the ε2ε3 genotype or ε2 allele had significantly higher SUA levels than ε3ε3 genotype or ε3 allele carriers. To the best of our knowledge, this is the first study to examine the possible associations between APOE polymorphism and hyperuricemia in China.

APOE gene polymorphism is known to be closely related to cardiovascular disease, hypertension, dyslipidemia, diabetes, and metabolic syndrome [28]. Hyperuricemia is an independent risk factor for coronary artery disease(CAD), hypertension, and obesity-related metabolic syndrome [29]. Therefore, it is reasonable to explore the connections between APOE gene polymorphism and hyperuricemia. Cardona et al. reported that the prevalence of the ε2 allele was greater in the patients with gout than in the healthy subjects and proposed that the reduced renal excretion of uric acid in patients with gout is mediated by the high prevalence of the ε2 allele of the APOE gene [19]. Another study in Caucasians also suggested that the APOE-ε2 allele is independently associated with increased SUA levels in healthy individuals [20]. In addition, according to various studies, the association between the ε4 allele and serum uric acid levels is controversial. Different studies have shown that APOE-ε4 allele carriers have lower [21], [22] or higher [23] SUA levels compared with APOE-ε3 allele carriers.

In this Chinese population study, the frequencies of the ε2ε3 genotype and the ε2 allele in hyperuricemic individuals were significantly higher than those in normouricemic subjects, but no difference in the frequency of the ε4 allele was found between the two groups. We further analyzed the associations of gender and ethnicity with APOE polymorphism, and we found that the APOE ε2 allele frequency was also significantly higher in the hyperuricemic group compared with the normouricemic group, not only in males and females but also in the Han and Hui populations(Table 3 and Table 4). Interestingly, we observed that the prevalence of the APOE ε2 allele in males was higher than that in females in both the hyperuricemic (11.3% vs. 8.8%) and normouricemic (6.7% vs. 4.8%) groups (Table 3), which may be another reason besides differences in sex hormones for the reduced renal excretion of uric acid and the high incidence of hyperuricemia in males. Denzer et al. reported a significantly positive association between plasma testosterone and uric acid concentrations in obese children and adolescents [30]. In addition, animal studies have demonstrated gender differences in uric acid transporters and showed that male mice have a higher reabsorption of uric acid than female mice [31]. The higher APOE ε2 allele frequency in men and its possible impact requires further confirmation and clarification. The frequencies of the APOE alleles are highly variable among different ethnic populations; for example, the ε4 allele frequency is significantly higher in African-American than in Asians [32]. Therefore, in this study, we explored the difference in the frequencies of APOE gene polymorphisms between Han and Hui populations, but we did not find any significant difference between the two groups.

A logistic regression model demonstrated that the ε2ε3 genotype and the ε2 allele were significantly positively associated with hyperuricemia, and the association remained significant after adjustment for gender, BMI, DBP, TG, LDL-C, FBG and Cr. The ε2ε3 genotype or ε2 allele could be considered as independent risk factors of hyperuricemia. The underlying mechanisms for the increased risk of hyperuricemia in individuals with the ε2ε3 genotype or the ε2 allele are largely unknown, but they are most likely related to increased purine metabolism and uric acid production or decreased renal clearance of uric acid. The presence of the APOE-ε2 allele in patients with gout is associated with reduced renal excretion of urates [19]. In fact, in this study, the positive association between the ε2 allele and hyperuricemia persists after excluding the impact of serum creatinine (SCr), suggesting that the main reason is not reduced renal clearance of uric acid, but more likely increased production of uric acid. The presence of APOE gene polymorphism has been demonstrated to influence serum levels of C-reactive protein (CRP) [33]. Moreover, epidemiological studies have confirmed that children with high concentrations of CRP are more susceptible to hyperuricemia, and the serum uric acid concentration increased with rising CRP levels [34]. Whether the APOE-ε2 allele could stimulate uric acid production and the underlying mechanisms require further experimental exploration. In this study, we did not find that BMI and DBP were independently associated with hyperuricemia (Table 7), probably because of the special nature of the study population. Patients included in this study are patients with primary hyperuricemia, among whom hypertension, diabetes and other diseases have been ruled out. In addition, teenagers are the main body of the study population. Therefore, in this population, it is possible that ApoE genotype, but not BMI and DBP, is independently associated with hyperuricemia.

Dietary habits, the intensity of physical activity, drinking and smoking may also be associated with the serum uric acid level. However, we did not include these factors in the multivariable analysis, which is a limitation of our study. In addition, due to the small sample size for subjects with the ε2ε2, ε2ε4 and ε4ε4 genotypes, these genotypes were not included in the logistic regression analysis.

In conclusion, this study is the first to report that the APOE ε2 allele is positively associated with SUA and could be an independent risk factor for hyperuricemia in a Chinese population. Larger studies in the future will further clarify the correlation between APOE polymorphisms and uric acid metabolism.
